# Phenotypic and molecular responses of copepods to UV radiation stress in a clear versus a glacially turbid lake

**DOI:** 10.1111/fwb.13953

**Published:** 2022-06-06

**Authors:** Barbara Tartarotti, Ruben Sommaruga, Nadine Saul

**Affiliations:** ^1^ Lake and Glacier Research Group Department of Ecology University of Innsbruck Innsbruck Austria; ^2^ Molecular Genetics Group Institute of Biology Humboldt University of Berlin Berlin Germany

**Keywords:** alpine lakes, glacier retreat, *hsp70*, photoprotection, zooplankton

## Abstract

1. Zooplankton are exposed to multiple environmental stressors in alpine lakes. However, phenotypic and molecular responses of copepods to different environmental conditions, including ultraviolet radiation (UVR), are still not fully understood. Here, we tested whether gene expression patterns vary within the same species, *Cyclops abyssorum tatricus*, but in populations from different environments (a clear vs. a glacially turbid lake) when exposed to UVR. Moreover, we wanted to examine potential seasonal variation (summer vs. autumn) in copepod gene expression.
2. We measured photoprotective compounds (mycosporine‐like amino acids and carotenoids) and antioxidant capacities in two copepod populations and studied gene expression of heat shock proteins (*hsp*s) as indicator of stress after UVR exposure in the laboratory.
3. Compared with the copepod population from the clear lake, the population from the turbid lake showed lower mycosporine‐like amino acid, but higher carotenoid concentrations that decreased over the season. Antioxidant capacities (both lipophilic and hydrophilic) were higher in autumn than in summer. The *hsp60* and *hsp90* genes were constitutively expressed, regardless of habitat origin and season, while *hsp70* was upregulated after exposure to UVR (up to 2.8‐fold change). We observed stronger upregulation of *hsp70* gene expression in autumn for the turbid and summer for the clear lake, with highest gene expression 24 hr post‐UVR exposure (up to 10.2‐fold change in the turbid and 3.9‐fold in the clear lake).
4. We show how variation in phenotypic traits modulates *hsp* gene expression patterns, specifically *hsp70* gene expression. Rapidly induced defences against cellular stress may improve survival in harsh environments such as alpine lakes, especially since these sensitive ecosystems may experience further changes in the future.

## 1 INTRODUCTION

Zooplankton such as copepods occupy an intermediate trophic position in the aquatic food web of most lake ecosystems (Lampert & Sommer, 2007). They inhabit lakes with a broad range of physicochemical and optical properties and are thus exposed to a variety of environmental threats and stressors that require specific responses. To survive and succeed under highly variable conditions, the animals adjust e.g. their biochemistry, physiology, morphology, behaviour, or life history (DeWitt et al., 1998; West‐Eberhard, 2003). On the smallest timescale, organisms activate an array of immediate stress responses (Lauritano et al., 2012).

Physiological adjustments are of particular importance in harsh environments such as alpine lakes (i.e., located above tree line), where plankton organisms face oligotrophy, low temperatures, and strong seasonal contrasts. Their number currently increases rapidly in mountain areas such as the Alps due to glacier retreat (Mölg et al., 2021), a phenomenon observed worldwide (Carrivick & Tweed, 2013). Newly formed lakes and those fed by glacial meltwaters show significant levels of turbidity, although these lakes will eventually turn clear when the hydrological connectivity to the glacier is lost (Desloges, 1994; Vinebrooke et al., 2010). Climate change‐induced shorter periods of ice cover will lead to earlier exposure to high levels of solar ultraviolet radiation (UVR) (Adrian et al., 2009), which significantly structures ecosystem processes in clear alpine lakes (Rose et al., 2009; Sommaruga, 2001). However, in the presence of glacier‐derived turbidity, the UVR threat is strongly reduced via attenuation of solar radiation (Rose et al., 2014; Tartarotti et al., 2017).

We have recently shown that copepods rely on a combination of behavioural and physiological strategies when lakes shift from glacially turbid to clear conditions (Tartarotti et al., 2017). In clear lakes, the highly energetic short wavelengths of solar UVR penetrate deep into the water column, resulting in potentially harmful effects on aquatic biota (see Rautio & Tartarotti, 2010 for a review).

In habitats where zooplankton cannot avoid hazardous radiation intensities, they rely on the accumulation of photoprotective compounds such as mycosporine‐like amino acids (MAAs) that directly screen UVR (Karentz et al., 1991) or antioxidants such as carotenoids that protect the cells by quenching reactive oxygen species (Cockell & Knowland, 1999). High concentrations of MAAs in alpine copepods (Persaud et al., 2007; Tartarotti et al., 2001; Tartarotti et al., 2004; Tartarotti et al., 2017) and their relation to lake elevation and UVR transparency (Tartarotti et al., 2001, 2004, 2017) support the idea that MAAs are essential for the survival of these organisms. The accumulation of carotenoids in copepods, a widely observed phenotypic adaptation, has also been linked to UVR‐induced photoprotection (Hairston Jr., 1979; Hansson, 2000; Ringelberg et al., 1984; Sommaruga, 2010; Tartarotti et al., 2018). However, observations in low‐UVR environments suggest that other factors such as reproduction and lipid metabolism may also control carotenoid accumulation (Schneider et al., 2012; Schneider et al., 2016; Sommer et al., 2006). Previous studies in clear lakes have revealed seasonal patterns underlying copepod content of MAAs and carotenoids (Moeller et al., 2005; Tartarotti et al., 2018; Tartarotti & Sommaruga, 2006). However, whereas MAAs concentrations are higher in copepods from clear than from turbid alpine lakes (i.e., positively linked to UVR exposure), carotenoid contents do not necessarily differ and may even show the opposite trend (Tartarotti et al., 2017).

As key components of the cellular stress response, heat shock proteins act as molecular chaperones and in the recovery of cells from stress by maintaining the integrity of cellular proteins (Feder & Hofmann, 1999; Sørensen et al., 2003). In aquatic organisms, a wide array of environmental challenges, ranging from thermal, oxidative, chemical to UVR stress, induces the expression of stress proteins (Feder & Hofmann, 1999; Sanders, 1993; Tomanek, 2010). Induction of *hsp* genes after exposure to UVR was observed in marine copepods (Kim et al., 2015; Won et al., 2015) and was found to be species‐ as well as gene‐specific (Han et al., 2016). Less information is available for freshwater copepods, but, recently, we have shown that the seasonal plasticity in photoprotection modulates UV‐induced *hsp* gene expression in cyclopoid copepods (Tartarotti et al., 2018). In the clear study lake, the highest expression levels (*hsp70*) were observed at times of low photoprotection (i.e., ice cover season).

Previous studies with freshwater copepods have found that not only the content of photoprotective compounds (Tartarotti et al., 2017), but also the levels of stress protein genes (*hsp60*, *hsp70*, and *hsp90*) (Tartarotti et al., 2019) differ in populations from either clear or glacially turbid lakes. Here, our aim was to test whether gene expression patterns vary within the same species but in populations from different environments when experimentally exposed to ecologically relevant levels of UVR. We sampled zooplankton from two alpine lakes differing largely in their physicochemical characteristics during summer and autumn to assess population‐specific and temporal variation in photoprotection levels and to determine how stressful UVR is using *hsp* gene expression as a proxy for stress.

## 2 METHODS

### 2.1 Study sites and study species

We collected the cyclopoid copepod *Cyclops abyssorum tatricus* Kozminski from the clear alpine lake Gossenköllesee (GKS) and from the turbid glacier‐fed lake Faselfadsee 3 (FAS3; Table 1 for lake description). We are aware that our study relies on one lake per lake type (clear/turbid); however, logistical constraints made it difficult to include replicate lakes within the clear and turbid lake category in this Alpine region. Samples were taken by several vertical net (50‐μm mesh) tows made at the centre of the lakes. Net tows were made from *c*. 1 m above the sediment to the surface in Lake GKS as UVR reaches the bottom in this lake and as the copepods are distributed close to it during the day (Tartarotti et al., 1999). In Lake FAS3, tows were made from 13 m depth to the surface, which corresponds to the portion of the water column where sufficient copepods can be caught (Tartarotti et al., 2017). Copepod samples were taken during the ice‐free summer (August) and autumn (end of September/October) period. Live copepods were transported (insulated cool boxes) and maintained at in situ temperature conditions and fed with *Cryptomonas* sp. upon return (within a few hours of sampling) to the laboratory in Innsbruck. After 48 hr of acclimation, the UV exposure experiments were done.

**TABLE 1:** Main characteristics of the study lakes including elevation, lake area, maximum lake depth (*Z*_max_), mean specific (25°C) electrical conductivity (Cond), mean pH, mean dissolved oxygen (O_2_), mean chlorophyll *a* (Chl *a*), and water optical properties (dissolved organic carbon content (DOC), mean (minimum and maximum) turbidity, and depth of 1% of surface irradiance for 320 nm (*Z*_1%320_) UV

Lake	Elevation (m a.s.l.)	Area (km^2^)	*Z*_max_ (m)	Cond (μS/cm)	pH	O_2_ (mg/L)	Chl *a* (μg/L)	DOC (mg/L)	Turb. (NTU)	*Z*_1%320_ (m)
Gossenköllesee (GKS)										
14 Aug 2014	2,417	0.017	9.9	23.1	7.23	8.7	1.68	0.33	<0.5	17.67^a^
28 Sep 2014				23.2	7.29	6.6	n.a.	0.37	<0.5	17.67^a^
Faselfadsee 3 (FAS3)										
06 Aug 2014	2,414	0.021	17.0	40.0	7.37	10.1	2.29	0.19	4.4 (3.6–5.2)	2.48
14 Oct 2014				54.1	7.59	8.5	n.a.	0.18	1.1 (1.0–1.3)	5.92

Abbreviation: n.a., not available.

^a^ Profiles from 26 June 2014.

### 2.2 Study design

For the experiments, we selected copepodid CIII–CV life stages, because these developmental stages were present in high numbers at both sites and dates. Experiments were done as described in detail previously (Tartarotti et al., 2018). Copepods (60 copepods per sample, three replicates) were flash frozen to determine background levels of the *hsp* gene expression in *C. abyssorum tatricus* before the experiments started (hereafter time zero, *t*_0_). For the experiments, copepods were carefully transferred into quartz glass tubes (250 ml of 10‐μm mesh‐filtered lake water and *c*. 60 copepods per tube and in triplicates) and were exposed to UVR plus photo‐reactivating radiation (UV treatment) or to photosynthetically active radiation alone (PAR treatment; tubes covered with vinyl chloride foil; C.I. Kasei Co.). Four A‐340 Q‐Panel lamps (Q‐Panel) and two white daylight lamps (F36W/860; General Electric Lightning) were used, resulting in irradiance values of 1.4 W/m^2^ (integrated between 280 and 320 nm; see Sommaruga et al., 1996 for further details). Quartz tubes covered with aluminium foil served as dark control (D, control; triplicates). The experiments were run in an environmental chamber set at ambient temperature (6–8°C; depending on the variation in the average water temperature on the day of sampling) for an exposure period of 6 hr. This period was chosen to simulate realistic exposure conditions in terms of duration and UVR dose. Copepodid to adult *C. abyssorum tatricus* are still experiencing *c*. 10% and *c*. 23% of the surface UV‐B (305 nm) and UV‐A (340 nm) irradiance in clear lake GKS during the ice‐free period under natural solar radiation conditions (Sommaruga & Psenner, 1997; Tartarotti & Sommaruga, 2006) despite their deep daytime distribution (Tartarotti et al., 1999), whereas the same species shows a more even vertical distribution or a preference for the middle to deeper water depths in turbid lakes (Tartarotti et al., 2017). At the end of the exposure period, copepods were placed in the dark for 1 hr (recovery period after relief of stress; Rhee et al., 2009) before they were flash frozen. Another set of animals (triplicates per UV and PAR treatment and dark control for FAS3) was kept in dark conditions for 24 hr to follow the post‐UV exposure stress response (post‐UV exposure). As there were not sufficient copepods from the GKS population available, only animals from the UV treatment (triplicates) were kept in dark conditions following UV exposure (24 hr post‐UVR exposure). Copepods from all treatments were checked for mortality under a stereo microscope (Olympus SZ40, Tokio, Japan), flash frozen in liquid nitrogen (starting with the UV treatment; sorting ≤10 min per sample) and stored at −80°C until the extraction of RNA.

### 2.3 Laboratory and field methods

#### 2.3.1 Mycosporine‐like amino acids, carotenoids, and antioxidant capacities

For antioxidant capacity measurements, copepods (copepodid CIII–CV life stages; 60 copepods per sample; triplicates) were flash frozen in liquid nitrogen (storage at −80°C) upon return to the laboratory at the University of Innsbruck within a few hours of sampling. Copepods for the analyses of MAAs and carotenoids were kept in the dark at 6–8°C and processed within 24 hr. The copepods were narcotised with CO_2_ and 10–12 (MAAs) and 30 (carotenoids) individuals (mostly copepodid CIII–CV life stages, no nauplii) were separated into microcentrifuge tubes. The samples (triplicates) were immediately frozen at −80°C.

We measured antioxidant capacities as described previously (Tartarotti et al., 2014). Briefly, the copepods were cleaved in a Speedmill (Analytik Jena, Jena, Germany) followed by centrifugation (11,600 g, 3 min) using a sodium hydrogen phosphate buffer (0.1 m, pH 6.5). The cooled supernatant was directly used to determine the antioxidant capacity of water‐soluble antioxidants or was further processed for the extraction of lipid‐soluble antioxidants following Bligh and Dyer (1959). We analysed antioxidant capacities based on Popov and Lewin (1999) in a PhotoChem device (Analytik Jena) via photo‐chemiluminescence. Copepod protein content was measured according to Bradford (1976), and antioxidant capacities were expressed as nm trolox or ascorbic acid equivalents/[mg protein] for lipophilic and hydrophilic antioxidants, respectively.

We extracted MAAs according to Tartarotti et al. (2017). Briefly, extraction was done in 400 μl of aqueous methanol (25% v/v; MeOH) in a water bath for 2 hr at 45°C. Samples were sonicated on ice at the beginning of the extraction (30 s at 40 W; UP 200S; Dr Hielscher GmbH) and stored overnight at −80°C for characterisation using isocratic reverse‐phase high performance liquid chromatography (HPLC; Dionex). To separate and quantify MAAs, aliquots (80 μl) were injected into a Phenosphere 5 μm RP‐8 column (4.6 mm internal diameter × 25 cm; Phenomenex) protected with a RP‐8 guard column (Brownlee, Perkin Elmer). The mobile phase of 0.1% acetic acid in 25% aqueous MeOH (v/v) had a flow rate of 0.75 ml/min. We used online UV spectroscopy in a Dionex poly‐diode array detector to detect the MAAs in the eluate. Identification of individual peaks was based on relative retention time, absorption spectra, and co‐chromatography with known standards (*Porphyra tenera*). We then calculated the content of specific MAAs in each sample from peak areas at 310, 320, 334, and 360 nm, using published molar extinction coefficients (see Tartarotti et al., 2001). MAA concentrations were normalised to the dry weight (DW) of the copepods and were expressed as [μg/mg DW]. For DW estimation, the *C. abyssorum tatricus* body length was measured at a magnification of 100× and biomass was then calculated according to Praptokardiyo (1979).

Carotenoids were extracted in 400 μl 95% ethanol at 8°C for 14 hr. The samples were sonicated on ice for 1 min (40 W) at the beginning of the extraction. Carotenoids were analysed using a double‐beam spectrophotometer (Hitachi U‐2000) against an ethanol blank. The concentration of carotenoids was calculated as in Hairston Jr. (1978) and expressed as [μg/mg DW].

#### 2.3.2 Extraction of RNA and cDNA synthesis

RNA extraction and cDNA synthesis were done as described in detail previously (Tartarotti et al., 2018). We used TRIzol reagent (Invitrogen) to extract total copepod RNA following the manufacturer’s instructions. We homogenised the samples and added gDNA eliminator solution (Qiagen) to eliminate potential genomic DNA contamination. The RNA extract was further purified (RNeasy Mini Kit, Qiagen; according the manufacturer’s protocol). Total RNA was measured (NanoDrop ND1000, Thermo Fisher Scientific) to assess RNA quality (mean A260/280 ratio: 2.10 ± 0.06) and run on a 1.2% agarose gel to assess RNA integrity. We quantified the RNA concentration in triplicate with a plate reader (2030 Multilabel Plate Reader Victor X4; Perkin Elmer) and the Quant‐iT RiboGreen Assay Kit (Life Technologies). Copepod RNA (450 ng) and random hexamer primers (#S0142; Thermo Scientific) were used for first‐strand complementary DNA (cDNA) synthesis. Following the manufacturer’s protocol, we synthesised cDNA using MMLV H minus reverse transcriptase (#EP0452; Thermo Scientific) with slight modifications by using less reverse transcriptase (100 U in 50‐μl reaction volume). We included no‐reverse‐transcriptase controls to test for potential genomic DNA contamination. Before quantitative polymerase chain reaction (qPCR) analysis, cDNA was frozen (−20°C).

#### 2.3.3 Quantitative polymerase chain reaction

We used oligonucleotide primers (hsp60 forward [f]: GGCTGGAGACGGTACCACAA, reverse [r]: ACCTGCCTTGGCAATTGC; efficiency [e]: 92%; hsp70 f: CAACCAGAAGCAGGGAAAGAAG, r: CCACCCCCGAGGTCAAA; e: 96%, and hsp90 f: AACATCAAGCTTGGTATCCATGAA, r: GAGGAGCCCGGCTAACTTCT; e: 96%) and qPCR conditions as described previously (Tartarotti et al., 2018). Briefly, qPCR reactions (triplicate technical repeats) were run in a QuantStudio3 real‐time PCR detection system (Thermo Fisher Scientific), using a mixture comprising 1× PowerSybr Green PCR Master Mix (Thermo Fisher Scientific), forward and reverse qPCR‐specific primer (500 nm each), nonacetylated bovine serum albumin (Sigma‐Aldrich), and cDNA. No‐reverse‐transcriptase controls were included. The qPCR conditions were 50°C/2 min; 40 cycles of 95°C/15 s, 60°C/1 min. Data acquisition and analysis were done with the QuantStudio™ Design and Analysis Software v1.4.1 (Thermo Fisher Scientific). Serial dilutions of gene‐specific quantified *C. abyssorum tatricus* cDNA were made for the determination of real‐time PCR efficiency. We calculated absolute copy numbers (absolute quantification method) by plotting the CT values versus the log10 of the initial copy numbers, quantified with the Quant‐iT Picogreen dsDNA Assay Kit (Life Technologies), and the specific molecular weight of each amplicon. The copy numbers were normalised to 10 ng of total copepod RNA. PCR products were subjected to melt‐curve analysis after amplification.

#### 2.3.4 Physicochemical parameters, chlorophyll *a*, and UV attenuation measurements

Before sampling, profiles for water temperature, electrical conductivity, dissolved oxygen, and pH were made using an YSI Exo2 probe (Xylem Inc.). For turbidity and chlorophyll *a* (Chl *a*) measurements, we collected water samples with a modified 5 L Schindler‐Patalas sampler from surface to the maximum depth (1–2 m depth intervals; depth integrated Chl *a* sample in FAS3). Turbidity was measured as nephelometric turbidity units (NTU) using a WTW Turb 430 T (Weilheim, Germany) calibrated between 0.02 and 1,000 NTU in the FAS3 samples. For Chl *a*, water samples of 0.5–1.1 L (prefiltered by a 100‐μm mesh to remove zooplankton) were filtered through glass fibre filters (GF/F, Whatman; pre‐combusted at 450°C for 2 hr) and the pigments were extracted in alkaline acetone. The equation of Lorenzen (1967) was used to calculate the Chl *a* concentration. Dissolved organic carbon (DOC) was measured in the filtrate (composite water sample; FAS3: 0–16 m depth, GKS: 0–9 m) with a total carbon analyser (Shimadzu TOC‐V_CPH_). In both lakes, underwater irradiance‐depth profiles were taken with a PUV‐501B profiler radiometer (Biospherical Instruments). The *K*_d_ in the water column was determined from the slope of the linear regression of the natural logarithm of down‐welling irradiance (*E*_d_) versus depth (*z*).

#### 2.3.5 Data analysis

We report all data as mean ± standard error; level of significance was set to *p* < 0.05. The significance of differences in photoprotection levels and *hsp* gene expression was evaluated by two‐way analysis of variance (ANOVA) with an interaction term between the explanatory factors *population* and *season* (for the response variables *MAA concentration*, *carotenoid concentration*, and *antioxidant capacities*) or *treatment* and *season* (for the response variables in the gene expression study), respectively, corrected for temporal autocorrelation via the Newey–West method and followed by Tukey HSD (honestly significant difference) post hoc test. For the *hsp* genes, copy numbers were log (natural logarithm, ln) transformed. We ran all statistical analyses using the software package XLSTAT (Version 1 March 2021).

## 3 RESULTS

### 3.1 Lake conditions

Turbidity strongly decreased in glacier‐fed Lake FAS3 from summer to autumn, resulting in a *c*. 2.4 times greater Z_1%_ depth in the latter season (Table 1), while turbidity is <0.5 NTU in clear GKS (personal observation). The water temperature ranged from 11.2 (surface) to 5.6°C (close to the bottom; summer) and from 9.2 to 8.0°C (autumn) in GKS, while temperatures were generally lower in the turbid lake (FAS3: range 6.3–5.0 and 5.5–5.3°C). In both lakes, the water column mixed during the autumn sampling.

### 3.2 Mycosporine‐like amino acid, carotenoid, and antioxidant capacities

**FIGURE 1:**
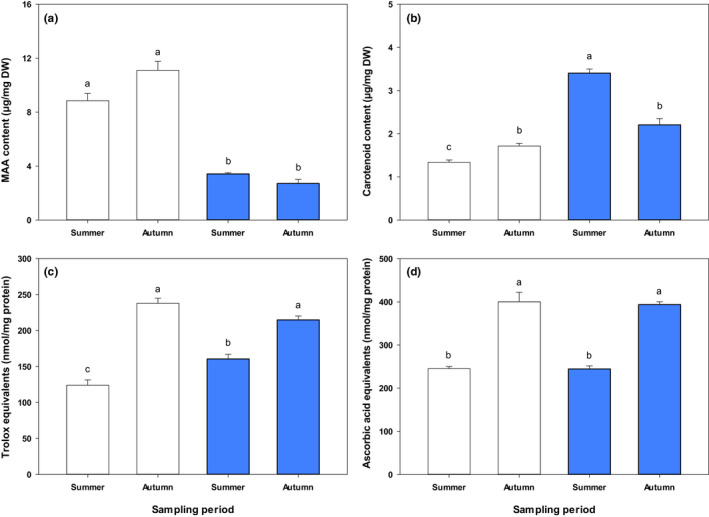
Variation of (a) total mean MAA and (b) carotenoid concentrations (μg/mg dry weight [DW]; *n* = 3), and (c) mean lipophilic (nmol trolox equivalents/mg protein; *n* = 3) and (d) hydrophilic antioxidant capacity (nmol ascorbic acid equivalents/mg protein; *n* = 3) in *Cyclops abyssorum tatricus* from the clear Lake Gossenköllesee in August and end of September 2014 (left; white bars) and from the turbid Lake Faselfadsee 3 in August and October 2014 (right; blue bars). Error bars indicate + *SE*. Different letters above the bars indicate a significant difference found with two‐way analysis of variance (ANOVA) with interactions followed by a Tukey HSD post hoc test

In FAS3, copepod carotenoid concentrations were significantly higher (*p* < 0.0001) in summer than in autumn, while there was no significant temporal change (*p* = 0.256) in the MAA contents (Figure 1a,b). The opposite trend was observed in the GKS population, where carotenoid contents were significantly higher (*p* = 0.011) in autumn than in summer. Concentrations of MAAs were also higher, but not significantly (*p* = 0.182) different in autumn. In both populations, antioxidant capacities were significantly lower (lipophilic metabolites: FAS3, *p* = 0.003; GKS, *p* < 0.0001; hydrophilic metabolites: FAS3, *p* < 0.0001; GKS, *p* = 0.001) during the summer sampling as compared with the autumn sampling (Figure 1c,d). When comparing the two populations, MAA levels were 2.6‐ (*p* < 0.0001; summer) and 4.1‐fold (*p* < 0.0001; autumn) higher in the copepods from GKS than in those from FAS3, whereas carotenoid concentrations were 2.5‐ (*p* < 0.0001) and 1.3‐fold (*p* = 0.054) higher in FAS3 than in GKS (Figure 1a,b). Lipophilic antioxidant capacities showed significant variation with higher values in the FAS3 than in the GKS population during summer (*p* = 0.024). In autumn, higher values, although not statistically significant (*p* = 0.126), were found in the GKS population (Figure 1c). Hydrophilic antioxidant capacities were almost identical between the *C. abyssorum tatricus* populations in both summer and autumn, respectively (*p* > 0.05; Figure 1d).

### 3.3 Expression of *hsp60*, *hsp70*, and *hsp90* genes

**FIGURE 2:**
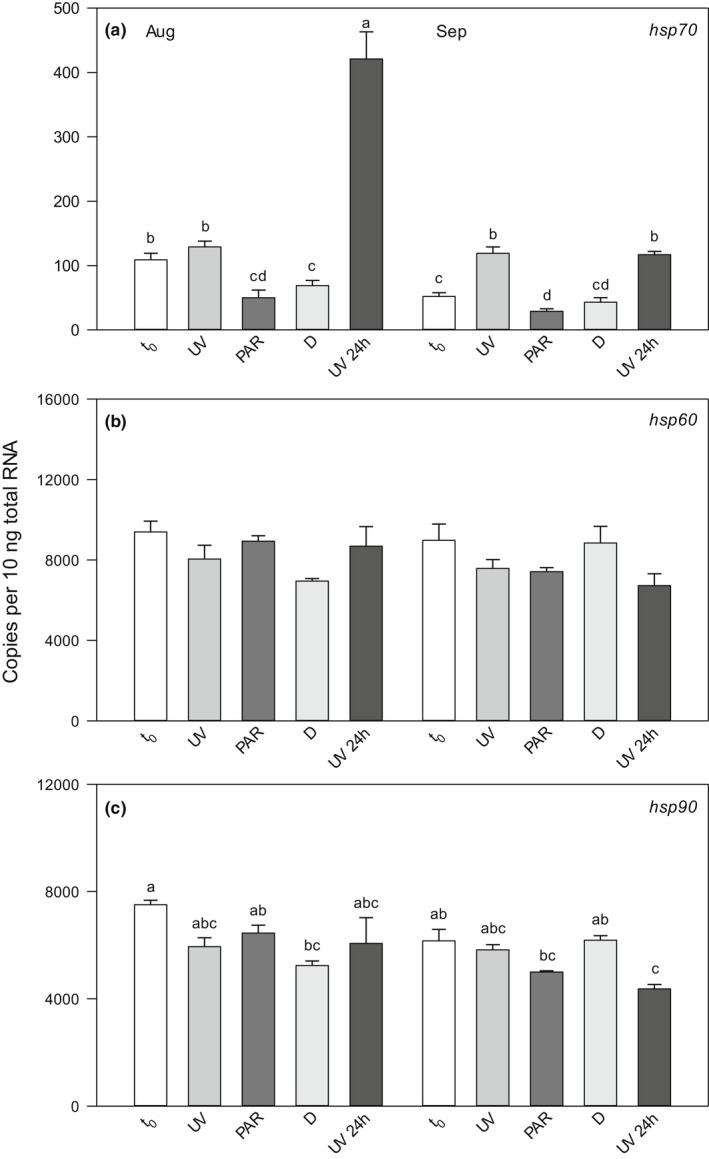
Expression of heat shock protein 70 (*hsp70*) gene (a), heat shock protein 60 (hsp60) gene (b) and heat shock protein 90 (*hsp90*) gene (c) in *Cyclops abyssorum tatricus* from the clear Lake Gossenköllesee in August and end of September 2014. Gene expression was quantified (absolute quantification method) at the beginning of the experiment (t_0_), following 6 hr of ultraviolet (UV) radiation exposure with photo‐reactivation radiation (UV), 6 hr of photosynthetically active radiation (PAR), when kept in the dark (d), and 24 hr post‐UV radiation exposure (UV 24 hr); *n* = 3 biological replicates, 60 copepodid CIII–CV life stages were pooled per sample. Shown are mean ± SE expression. Different letters above the bars indicate a significant difference found with two‐way analysis of variance (ANOVA) with interactions followed by a Tukey HSD post hoc test

**FIGURE 3:**
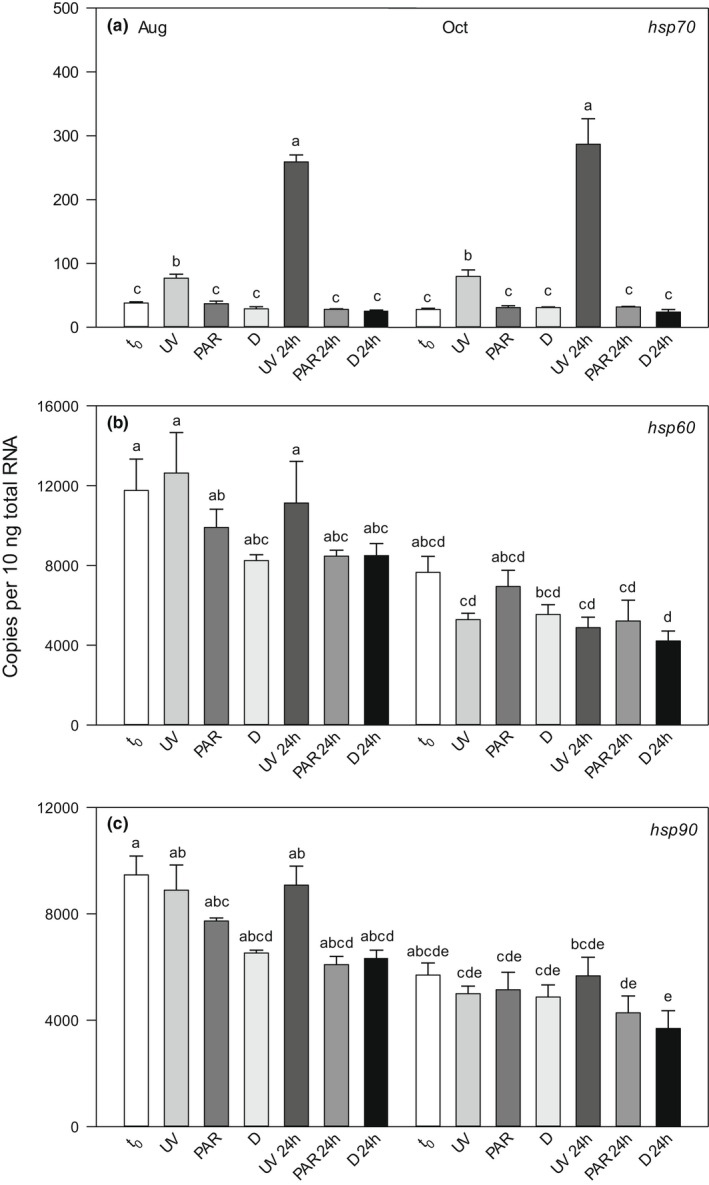
Expression of heat shock protein 70 (hsp70) gene (a), heat shock protein 60 (hsp60) gene (b) and heat shock protein 90 (hsp90) gene (c) in *Cyclops abyssorum tatricus* from the turbid Lake Faselfadsee 3 in August and October 2014. Gene expression was quantified (absolute quantification method) at the beginning of the experiment (*t*_0_), following 6 hr of ultraviolet (UV) radiation exposure with photo‐reactivation radiation (UV), 6 hr of photosynthetically active radiation (PAR), when kept in the dark (d), and 24 hr post‐UV exposure (UV 24 hr, PAR 24 hr, and D 24 hr); *n* = 3 biological replicates, 60 copepodid CIII–CV life stages were pooled per sample. Shown are mean ± *SE* expression. Different letters above the bars indicate a significant difference found with two‐way analysis of variance (ANOVA) with interactions followed by a Tukey HSD post hoc test

Copepod mortality was negligible in the different exposure treatments and the dark control, except for the UV treatment from FAS3 in autumn (mortality *c*. 10%). It was also in this treatment that a reduced motility and escape response as compared with the other treatments/control was observed. Even though the three *hsp* genes were expressed in all samples, only the gene expression of *hsp70* increased significantly in response to UVR exposure (Figures 2 and 3). Both the *hsp60* and *hsp90* gene expression showed some variation between the experimental treatments. However, there was no consistent pattern of up‐ or downregulation in a specific treatment and at no time was an induction of these genes observed after UVR exposure (Figures 2b,c and 3b,c). For both populations and seasons, highest *hsp70* gene expression was found in the UV and 24 hr post‐UV exposure treatments, with maxima in the latter treatment (Figures 2a and 3a).

### 3.4 Relationship between photoprotection and *hsp* gene expression

Relative to the gene expression at the beginning of the experiment (*t*_0_), the extent of *hsp70* upregulation after UVR exposure was highest (2.8‐fold; *p* < 0.0001) in the copepod population from turbid FAS3 at times of lower photoprotection (i.e., autumn; Figures 1b and 3a). During summer, when the copepods had higher pigment concentrations, it was 2‐fold (*p* < 0.0001) and more similar to the population from clear GKS (summer 1.2‐ and autumn 2.3‐fold upregulation; *p* > 0.05 and *p* < 0.0001, respectively; Figure 2a). In both populations, the strongest upregulation of gene expression was found in the 24 hr post‐UV exposure treatment (3.9‐fold for GKS in summer, *p* = 0.001; 10.2‐fold for FAS3 in autumn, *p* < 0.0001; Figures 2a and 3a). Even when the copepods had higher photoprotection levels (summer in FAS3 and autumn in GKS), the response was strong in the 24 hr post‐UV treatment in the turbid lake (6.9‐fold upregulation, *p* < 0.0001), but less pronounced in the clear one (2.2‐fold, *p* < 0.0001).

## 4 DISCUSSION

The study systems were similar in many ways, but differed mainly in turbidity, but also in maximum depth or electrical conductivity, as well as in thermal properties and the presence of fish. We did not aim to separate these potentially confounding effects, of which several are linked to the influence of a glacier in the catchment, but wanted to assess how variation in phenotypic traits of plankton organisms living in these ecosystems can be modulated by molecular mechanisms. We are aware that our data are based on two study sites and two populations, thus, the low number of lakes (*n* = 2) and replication limits extrapolation to the generality of the findings. Therefore, it cannot be excluded that the observed differences between the two lakes are not based on the turbidity. The occurrence of predators, the amount and quality of food or the presence of pollutants might be among the factors, which can differ between the lakes (Kammerlander et al., 2016; Laspoumaderes et al., 2017; Slemmons et al., 2013) and which could cause differences in the stress response. Thus, this study can only give a first indication and should motivate others to perform further investigations including additional sampling sites. Despite these caveats in the study design, the selected ecosystems are characteristic representatives of alpine lakes and *C. abyssorum tatricus* is a common and widely distributed copepod species in the Alps (Jersabek et al., 2001).

Autumn maxima in MAA and carotenoid contents seem to be typical for copepods from clear alpine lakes (Tartarotti et al., 2018; Tartarotti & Sommaruga, 2006). Our data indicate that populations from a turbid glacier‐fed lake show an earlier onset of the reduction in photoprotection, as specifically carotenoids decrease towards the end of the ice‐free season. This trend is similar to the one found in copepods from low‐UVR, boreal ecosystems, where pigment levels (mainly astaxanthin) decrease during spring/summer until they reach a minimum in autumn, which is followed by a maximum in winter (Schneider et al., 2016). Highest carotenoid values in winter have been reported in several studies (Ekvall et al., 2015; Hairston Jr., 1979; Hansson, 2004). However, as our observations are based on one summer and autumn sampling and one lake, further investigation is required to test if copepods from glacially turbid lakes follow this same seasonal pattern or reach minima in winter as observed in copepods from clear alpine lakes (Tartarotti et al., 2018). Interestingly, antioxidant capacities were higher in autumn than in summer in both tested copepod populations, which might impose more stressful conditions (e.g., overall lower water temperatures, mixing event) during that time of the year. However, this is in contrast to previous findings, which showed higher antioxidant capacities in the Lake GKS copepod population during summer (Tartarotti et al., 2018). While MAAs and carotenoids have to be accumulated and reflect longer‐term conditions (days to weeks; Moeller et al., 2005; Weaver et al., 2018), antioxidant metabolites are de novo synthesised and have the potential to change within hours (Borgeraas & Hessen, 2002; Tartarotti et al., 2019). Thus, antioxidant responses are rapidly activated at times when they may be needed the most to reduce the risk of oxidative damage.

One reason for the higher carotenoid levels in the population from the turbid lake may be that this ecosystem is, in contrast to Lake GKS, fish‐free. Several studies have shown the phenotypic plasticity of carotenoid levels in the presence of predators (Hansson, 2000, 2004; Hylander et al., 2012). Lower pigment concentrations are consistently reported for copepods when exposed to predation threat (Hansson, 2000). By contrast, even in fishless lakes carotenoid contents can be higher in a turbid than in a nearby located clear lake (Tartarotti et al., 2017). Surprisingly, carotenoid levels of cyclopoid copepods, unlike MAA concentrations, seem to be less influenced by suspended particles, as also observed in a previous study, which showed no clear relationship between carotenoids and lake transparency (Tartarotti et al., 2017). The high pigment concentrations of copepods in the turbid lake indicate that the role of these compounds as antioxidants is not only to protect organisms from damaging blue wavelengths/UVR as previously described for copepods from clear mountain lakes (Hairston Jr., 1978; Ringelberg et al., 1984), but that they have other physiological functions. Recent studies have shown that carotenoid accumulation is rather linked to lipid metabolism (antioxidant protection of storage lipids) and reproduction than UVR protection in low‐UVR environments (Schneider et al., 2016; Schneider et al., 2017). Alternative roles of carotenoids in copepods from turbid lakes are indirectly supported by significantly higher lipophilic antioxidant capacities in the FAS3 than in the GKS population during summer. These lipid‐soluble antioxidants include β‐carotene, which serves as precursor for the synthesis of astaxanthin in copepods (Andersson et al., 2003).

Levels of photoprotective compounds in the copepod population from the turbid lake decreased from summer to autumn, despite the strong increase in the depth of 1% of the surface irradiance at 320 nm (Z_1%320_). Although UVR penetrates deep into the water column of clear alpine lakes, planktonic organisms can avoid upper water layers by migrating to deeper waters (Alonso et al., 2004; Fischer et al., 2015; Rautio & Tartarotti, 2010). In fact, even within the same lake ecosystem, copepods stay deeper in the water column when glacial turbidity decreases (Tartarotti et al., 2017). One reason for our findings might be that maximum daily global total irradiance levels are already low in October, thus potential UV‐induced damage is minimised at that time of the year.

Despite population‐level differences in key traits related to UVR tolerance, our data based on two populations show that a specific heat shock response during stress exposure seems to be conserved within this species. Albeit the generally low photoprotection in the copepod population from the turbid lake, UVR effects at the molecular level are higher at times when the concentrations of protective compounds are even lower (i.e., autumn; 6.9‐ and 10.2‐fold *hsp70* expression change compared to t_0_ in summer and autumn, respectively). The maximum up‐regulation (*c*. 10‐fold) in *hsp70* gene expression in *C. abyssorum tatricus* is similar to the one reported for marine copepods (8‐fold change after UVR exposure; Kim et al., 2015) and to the one found in the copepod population from clear lake GKS at times of low protection during winter (9.6‐fold; Tartarotti et al., 2018). The lowest MAA concentration (3.88 μg/mg DW, under ice cover; data from Tartarotti et al., 2018) of the population from the clear lake was still higher (although not significantly; *t*‐test, *p* = 0.172) than the one from the turbid lake (2.72 μg/mg DW; this study), whereas carotenoid levels were significantly higher in the copepods from the turbid than from the clear lake (2.21 vs. 0.83 μg/mg DW; *p* = 0.002). Thus, in terms of photoprotection, higher concentrations of carotenoids do not seem to compensate lower MAA contents in our study and the protection of copepods from damaging UVR levels appears to rely mainly on MAAs in the two tested copepod populations. The question arises, if a tenfold increase in *hsp70* gene expression is the maximum in sublethal response, as reduced motility was observed in the UV‐exposed copepods and only in this treatment copepods started to die. Future studies should include short‐term stress responses in copepods when exposed in the field under natural environmental conditions.

Several studies have shown that not necessarily all *hsp* family genes respond in the same way. For example, heat stress induced *hsp70* but not *hsp90* expression in marine copepods (Rahlff et al., 2017; Rhee et al., 2009) and the induction of *hsp70* but not *hsp60* and *hsp90* genes was observed after UVR stress in *C. abyssorum tatricus* (Tartarotti et al., 2018; this study). Our data show that regardless of whether the copepods came from a high (clear lake) or low UVR environment (turbid lake), the response was similar, i.e. only constitutive levels of *hsp60* and *hsp90* gene expression were observed. It was reported recently hat the costly up‐ or down‐regulation of *hsps* does not necessarily occur on a daily basis in these organisms; however, population‐specific and seasonal differences in levels of stress proteins seem to be common (Tartarotti et al., 2018, 2019).

We are aware that the gene expression at the beginning of the experiments (*t*_0_) after acclimatisation to laboratory conditions might not be identical to the one observed in the natural population (baseline expression). However, previous findings show that *t*_0_ reflects the baseline expression well (Tartarotti et al., 2018), which allows comparison of gene expression levels between natural and acclimatised copepod populations. Recently, we have found that the mitochondrial *hsp60* gene was higher in copepods from a turbid than from a clear lake during the ice‐free summer period (Tartarotti et al., 2019). While our present study confirms this pattern for the summer sampling, expression levels decreased during autumn and higher levels were observed in the population from the clear lake at that time. Although copepods seem to be less affected by glacial mineral particles than cladocerans, effects of suspended sediments on vital rates (e.g. ingestion rate; Arendt et al., 2011) have also been reported for these selectively feeding animals (DeMott, 1986). As water turbidity in Lake FAS3 was four times lower in October than in August, the energy demand caused by, for example, changes in swimming behaviour in the presence of non‐ingestible particles as shown in marine copepods (Hansen et al., 1991) may decrease and may be reflected in the lower levels of constitutively expressed *hsp60* genes.

When turbid lakes become clear, as glaciers in the Alps disappear (Sommer et al., 2020), copepods will respond with changes in behaviour (i.e., staying deeper in the water column) and photoprotection (i.e., gradually increasing photoprotection levels). During this time, the potential to increase *hsp70* gene expression may help maintain protein homeostasis and provide immediate response when needed, thereby ensuring survival in harsh environments such as alpine lakes.

## AUTHOR CONTRIBUTIONS

B.T. conceived and designed the study; B.T. collected the samples; B.T. and N.S. analysed the samples; B.T. led the writing of the manuscript; R.S. and N.S. contributed to the draft; all authors approved the final manuscript.

## CONFLICT OF INTEREST

All authors declare no conflict of interest.

## Data Availability

The data that support the findings of this study are available from the corresponding author upon reasonable request.
